# Bioeconomy perception by future stakeholders: Hearing from European forestry students

**DOI:** 10.1007/s13280-020-01376-y

**Published:** 2020-10-13

**Authors:** Mauro Masiero, Laura Secco, Davide Pettenella, Riccardo Da Re, Hanna Bernö, Ariane Carreira, Alexander Dobrovolsky, Blanka Giertlieova, Alexandru Giurca, Sara Holmgren, Cecilia Mark-Herbert, Lenka Navrátilová, Helga Pülzl, Lea Ranacher, Alessandra Salvalaggio, Arnaud Sergent, Juuso Sopanen, Cristoph Stelzer, Theresa Stetter, Lauri Valsta, Jozef Výbošťok, Ida Wallin

**Affiliations:** 1 Department of Land, Environment, Agriculture and Forestry, University of Padova, Viale dell Università, 16 35020 Legnaro, PD Italy; 2 Swedish University of Agricultural Sciences, Ulls väg 27, 756 51 Uppsala, Sweden; 3 National Research Institute of Science and Technology for Environment and Agriculture, 50 Avenue de Verdun, 33612 Bordeaux, Cestas, France; 4 Saint-Petersburg State Forest Technical University, Institutskiy per 5, Saint-Petersburg, Russian Federation 194021; 5 Department of Economics and Management of Forestry, Faculty of Forestry, Technical University in Zvolen, T. G. Masaryka 24, 960 01 Zvolen, Slovakia; 6 Chair of Forest and Environmental Policy, University of Freiburg, Tennenbacherstr. 4, 79106 Freiburg, Germany; 7 Institute of Forest, Environmental and Natural Resource Policy, University of Natural Resources and Life Sciences (BOKU) in Vienna, Feistmantelstraße 4, 1180 Vienna, Austria; 8 Department of Forest Sciences, University of Helsinki, Latokartanonkaari 7, 00790 Helsinki, Finland; 9 Wood K plus – Competence Center for Wood Composites and Wood Chemistry, Kompetenzzentrum Holz GmbH, Altenberger Straße 69, 4040 Linz, Austria

**Keywords:** Bioeconomy, Education, Forest-based bioeconomy, Forestry students, Future stakeholders, Perception

## Abstract

This article provides useful information for universities offering forestry programs and facing the growing demand for bioeconomy education. An explorative survey on bioeconomy perception among 1400 students enrolled in 29 universities across nine European countries offering forestry programs was performed. The data have been elaborated via descriptive statistics and cluster analysis. Around 70% of respondents have heard about the bioeconomy, mainly through university courses. Students perceive forestry as the most important sector for bioeconomy; however, the extent of perceived importance of forestry varies between countries, most significantly across groups of countries along a North–South European axis. Although differences across bachelor and master programs are less pronounced, they shed light on how bioeconomy is addressed by university programs and the level of student satisfaction with this. These differences and particularities are relevant for potential development routes towards comprehensive bioeconomy curricula at European forestry universities with a forestry focus.

**Electronic supplementary material:**

The online version of this article (10.1007/s13280-020-01376-y) contains supplementary material, which is available to authorized users.

## Introduction

The transition to a bioeconomy can take many forms and will need wide social as well as political-institutional changes shaping possible future action (Goven and Pavone 2015) while requiring transformational efforts (Dietz et al. 2018; Lewandowski 2018). In order to face such changes and transformations, and to avoid that bioeconomy remains an “elite master narrative […that…] does not depend on popular acceptance, acquiescence and even awareness” (Birch et al. 2010, p. 2905), public opinions and social preferences shall be taken into account. For involving stakeholders at a societal level in discussions and decision-making processes, their perceptions about matters that directly affect their well-being need to be investigated (Mustalahti 2017). While a number of studies were conducted on bioeconomy, bio-based products, and associated technologies, research has only recently started to focus on interactions between actors involved in a bioeconomy (Hodge et al. 2017; Lewandowski 2018; Stein et al. 2018) and about the role of society in shaping and co-creating bioeconomy (Ramcilovic-Suominen and Pülzl 2018; Golowko et al. 2019; Sanz-Hernández et al. 2019).

Among future stakeholders, university students will be centre-stage for the development and implementation of a bioeconomy as future decision makers and a key future workforce shaping and enabling it. Already now the bioeconomy employs 8.2% of the European Union (EU) labour force (EC 2019), and is expected to stimulate new types of job opportunities, particularly within the forest-based sector, while the number of traditional forestry-related jobs continues to decrease (UNECE/FAO 2018). The potential to generate new (green) jobs through bioeconomy, however, also depends on universities’ ability to answer the demand for the interdisciplinary skills and specifically educated professionals needed for an innovative bioeconomy (Lewandowski 2018; UNECE/FAO 2018). In addition, educators have raised concerns over the ever-increasing shortage of talented workforce needed to realize ambitious bioeconomy goals. For example, Hakovirta and Lucia (2019) claim that the bioeconomy needs to attract youth to science, technology, engineering and mathematics disciplines while cultivating the multidisciplinary skills needed for a future workforce. The enhancement and updating of educational programs and learning initiatives were pointed out as important aspects for enabling bioeconomy and the associated societal transition (Lewandowski 2018; Golowko et al. 2019). In this regard, Higher Education Institutions (HEI) play central role as they are a prerequisite for transformational efforts by increasing competencies and facilitating transition via transparent, participative processes and a close dialogue across multiple disciplines (Herget 2018).

So far, few studies addressed students’ perception of bioeconomy (e.g. Drejerska 2017; Hempel et al. 2018; Stern et al. 2018a, b). Student-specific as well as cross-country comparative studies are rare (Mastalka and Timonen 2017; Golowko et al. 2019) and consider students’ perception from very specific angles (e.g. Pätäri et al. 2017). The research gap on bioeconomy perception among students is even more evident when focusing on forest-based bioeconomy (FBB) i.e. the segment of bioeconomy depending on forest resources as material/service providers for a transition to an alternative economic model (Scarlat et al. 2015). The “bioeconomy is expected to be the guiding paradigm within the forest-based sector in the years to come” (Wolfslehner et al. 2016, p. 5). Therefore, this explorative study aims to fill this research gap by investigating and comparing the current perceptions of bioeconomy by forestry students in different parts of Europe. In doing so, the following research questions are addressed:

1. To what degree have forestry students heard about bioeconomy and what are their sources of information?
2. How forestry students enrolled in different programs across different countries in Europe perceive bioeconomy and FBB? In particular:
  2.1 How do students perceive their information and training regarding bioeconomy at university?
  2.2 How do forestry students perceive the role of the forest sector within the bioeconomy today, both at the European and the national scale?
  2.3 What are the current and future FBB development drivers/orientations and possible impacts students perceive?

By addressing these questions, the paper aims to provide exploratory information that might be helpful for universities offering forestry-related programs interested to consider adapting and expanding their curricula. At the same time, by highlighting current bioeconomy perceptions of key future stakeholders, and analyse them comparatively, it aims to identify possible educational development trajectories that can be considered by policy and decision makers at both the national and European level.

The next section of the paper describes materials and methods employed, as well as the scope of the study. Results are presented in section three and discussed in section four. Finally, section five draws conclusions, highlighting the main findings, limitations and further research needs.

## Materials and methods

An explorative multi-language online questionnaire (Appendix S1) was developed targeted at Bachelor (BSc), Master (MSc) and Doctorate (PhD) students currently enrolled in forestry programs across Europe. Student programs were selected to cover all distinct European regions: Northern (Finland-FIN and Sweden-SWE), Central-Western (Austria-AUT, Germany-GER, France-FRA), Southern (Italy-ITA and Spain-ESP) and Eastern Europe (Slovakia-SVK and the Russian Federation-RUS). One or more top-ranked forestry universities from each country were selected for the survey. The opportunities for a FBB differ across the European continent (Hurmekoski et al. 2019) and therefore the selection of target countries is designed to capture this variation as they are also at different stages in their bioeconomy policy development. For example, GER and FIN have dedicated national bioeconomy strategies since a longer time period as compared to FRA, ITA and ESP. AUT published its strategy only in 2019, while SVK has just developed one and SWE and RUS have other bioeconomy-relevant policy initiatives in place.

The questionnaire was made available in a default English version as well as national languages. It consisted of open, close-ended, multiple choice and rating-scale questions organized into six sections. The present study focuses on the following three however:

1. “*Familiarity with bioeconomy”* investigated how familiar students are with the bioeconomy concept and related strategies at both European and national scale. Respondents were not given a definition of bioeconomy at this stage, as one of the aims of the survey was to investigate ‘blind’ knowledge and their perception.
2. “*Bioeconomy at university*” investigated to what extent and in which courses bioeconomy was addressed within university forestry programs. At the beginning of this section, the European Commission (EC 2012, 2018) definition for bioeconomy was provided.
3. “*Activities, issues, sectors, and actors associated to bioeconomy*” aimed at identifying students’ bioeconomy perception for Europe and the country they were studying in as well as expected bioeconomy barriers, divers and impacts. Special attention was paid to FBB and the role of the forest sector within bioeconomy.

The study relied on convenience sampling and was not intended to be statistically representative at the national scale, as data collection would have otherwise been too resource intensive. The aim was rather to reach as many forestry students as possible.

The questionnaire was developed based on a literature review, building on available examples of surveys on bioeconomy perceptions (e.g. Ranacher et al. 2017; Golowko et al. 2019) as well as on three rounds of expert feedback defined via brainstorming among authors (including students) to cover relevant topical aspects, including bioeconomy policy strategies.

The survey was pre-tested with students and amendments were made before final publication and launching. Data collection was done via Lime Survey between January and June 2019 with the support of MSc/PhD students in most of the countries. The survey was promoted via students mailing lists and social media. To increase success rate in data collection hard copy questionnaires were also distributed to students during different courses offered within forestry programs at hosting universities. Data were then manually transferred (though still kept identifiable) into the online survey system, in order to develop a common dataset.

A total of 1368 valid questionnaires was collected and used for data analysis. Table 1 and Appendix S2 summarize the distribution of valid questionnaires across the target countries and provide basic information on respondents.

**Table 1:** Respondents’ profile−Number and percentage of respondents per country, gender and study program

Respondents	AUT	ESP	FIN	FRA	GER	ITA	RUS	SVK	SWE	TOTAL
	216	68	61	21	237	329	81	225	130	1 368
*Of which (per gender)*										
Female	65	19	24	4	88	102	43	93	52	490
Male	142	47	32	17	147	223	36	131	74	849
N/A*	9	2	5	–	2	4	2	1	4	29
*Of which (per study program)*										
BSc	143	60	22	5	163	200	62	106	73	834
MSc	66	8	35	6	54	118	14	109	45	455
PhD	6	–	3	1	20	8	–	6	3	47
Other	–	–	–	7	–	3	1	–	3	14
Blank	1	–	1	2	–	–	4	4	6	18
*Respondents,* %	*15.8*%	*5.0*%	*4.5*%	*1.5*%	*17.3*%	*24.0*%	*5.9*%	*16.4*%	*9.5*%	*100.0*%
*Of which (per gender)*										
*Female,* %	*30.1*%	*27.9*%	*39.3*%	*19.0*%	*37.1*%	*31.0*%	*53.1*%	*41.3*%	*40.0*%	*35.8*%
*Male.* %	*65.7*%	*69.1*%	*52.5*%	*81.0*%	*62.0*%	*67.8*%	*44.4*%	*58.2*%	*56.9*%	*62.1*%
*N/A* %	*4.2*%	*2.9*%	*8.2*%	–	*0.8*%	*1.2*%	*2.5*%	*0.4*%	*3.0*%	*2.1*%
*Of which (per study program)*										
*BSc.* %	*66.2*%	*88.2*%	*36.1*%	*23.8*%	*68.8*%	*60.8*%	*76.5*%	*47.1*%	*56.2*%	*61.0*%
*MSc.* %	*30.6*%	*11.8*%	*57.4*%	*28.6*%	*22.8*%	*35.9*%	*17.3*%	*48.4*%	*34.6*%	*33.3*%
*PhD.* %	*2.8*%	–	*4.9*%	*4.8*%	*8.4*%	*2.4*%	–	*2.7*%	*2.3*%	*3.4*%
*Other.* %	–	–	–	*33.3*%	–	*0.9*%	*1.2*%	–	*2.3*%	*1.0*%
*Blank.* %	*0.5*%	–	*1.6*%	*9.5*%	–	–	*4.9*%	*1.8*%	*4.6*%	*1.3*%

*AUT* Austria, *ESP* Spain, *FIN* Finland, *FRA* France, *GER*  Germany, *ITA* Italy, *RUS* Russian Federation, *SVK* Slovakia, *SWE* Sweden, *N/A* not indicated or not reported

With reference to the first research question, statistical data analysis was conducted by means of Microsoft Excel for descriptive statistics. For the second research question, a hierarchical cluster analysis was performed via R version 4.0.0, using Orange 3.25 for data visualization. The Ward (1963) algorithm (minimum variance method) was used to measure dissimilarity: it allows the creation of a cluster at each step by including the observations that lead to the minimum increase in the intra-cluster variance. The initial distance between observations is defined by the squared Euclidean distance. We draw conclusions about the similarity of two observations based on the location on the horizontal axis where branches containing those observations are merged (James et al. 2013). The analysis was based on 23 variables associated to questions of the questionnaire and organized into three main blocks, i.e. one for each research sub-question (2.1, 2.2 and 2.3) (Table 2). In order to support the decision about the number of clusters in the dendrogram, a Gap statistic was considered (Tibshirani et al. 2001). The applied algorithm compares the change in within-cluster dispersion with its expected value under the null hypothesis (i.e. no clustering). The higher the Gap statistic, the better the clustering. This analysis showed that the best clustering in our dataset is obtained with 5 clusters.

**Table 2:** Variables used for the cluster analysis, their description, name in short and basic statistical values

Variables				Basic statistical values					
Block	Description		In short*	Median	Mean	SE mean	Var	Std.Dev	Coef.Var
2.1	Bioeconomy within attended university programs	Perception of the extent to which bioeconomy is currently addressed	S23	2.85	3.03	0.10	0.28	0.52	0.17
		Satisfaction with the extent to which bioeconomy is currently addressed	S24	2.55	2.52	0.10	0.28	0.51	0.20
		Extent to which bioeconomy should be addressed more within student’s university program	S25	3.12	3.12	0.13	0.42	0.63	0.20
2.2	Perceived current role of forests within bioeconomy	At European level	S35	4.16	4.17	0.07	0.14	0.36	0.09
		At country level	S37	4.00	4.10	0.11	0.32	0.55	0.13
2.3a	Aspects/issues developed through FBB nowadays	Totally new products & technologies	S39New	3.33	3.49	0.12	0.37	0.60	0.17
		Improvement of existing products	S39Imp	3.64	3.59	0.09	0.23	0.47	0.13
		Efficient use of forest-based product	S39Eff	3.79	3.79	0.06	0.11	0.32	0.08
		New uses for existing products	S39New	3.50	3.48	0.06	0.10	0.31	0.09
		Substitution of fossil fuels with forest biomass for energy purposes	S39Sub	3.58	3.65	0.11	0.32	0.56	0.15
		Multiple services/products offered by forests (e.g. ecosystem services)	S39Es	3.41	3.44	0.10	0.24	0.48	0.14
2.3b	Perceived FBB development drivers and orientations	Technological developments	S311Tec	3.53	3.51	0.11	0.33	0.56	0.16
		Oriented to products	S311Pro	3.44	3.51	0.06	0.11	0.32	0.09
		Oriented to multiple services	S311Ser	3.79	3.77	0.07	0.12	0.33	0.09
		Based on local natural resources	S311Lres	4.13	4.16	0.07	0.15	0.37	0.09
		Based on natural resources (no matter if local or imported)	S311Nres	3.00	2.94	0.11	0.29	0.53	0.18
		Combination of new and traditional knowledge	S311N&TK	4.14	4.11	0.07	0.13	0.35	0.09
2.3c	Perceived FBB development impacts	Promote employment opportunities	S311Emp	4.00	3.98	0.08	0.18	0.42	0.10
		Favour sustainable forest management (SFM)	S311SFM	4.16	4.07	0.11	0.31	0.55	0.13
		Promote FM at local scale	S311LoFM	3.84	3.84	0.09	0.23	0.47	0.12
		Promote FM, no matter at which scale	S311FM	3.59	3.68	0.09	0.20	0.43	0.12
		Increased deforestation/forest degradation	S311Def	2.33	2.25	0.11	0.30	0.54	0.24
		Increased people’s awareness of environmental and forestry issues	S311Awa	3.79	3.73	0.13	0.42	0.63	0.17

Variable values have been obtained from survey data by averaging values per country and attended program (i.e. BSC, MSc and PhD). See additional material available in Appendix S1 for more details and referenced questions

Data were analysed with regard to the whole sample and for single countries as well as university programs, in order to allow a comparative analysis and identify differences and similarities based on selected variables. The results are discussed *vis*-*à*-*vis* the existing literature on bioeconomy.

## Results

### To what degree have students heard about bioeconomy?

**Fig. 1:**
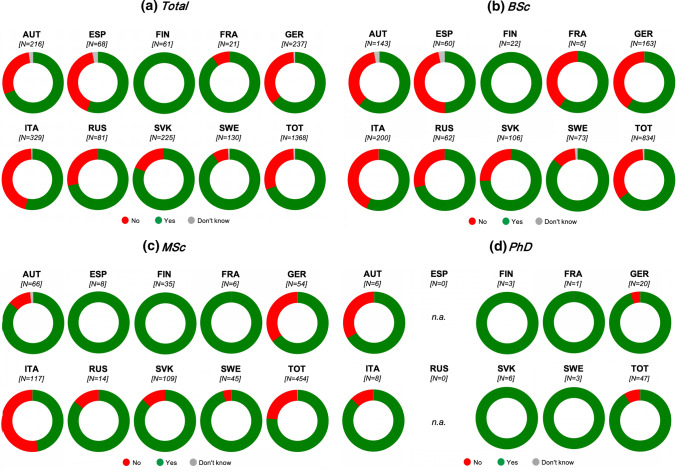
Respondents who have (yes)/haven’t (no) heard about bioeconomy: figures for all respondents and per attended program

About 70% of respondents have heard about bioeconomy: for all target countries the figure is higher than 50%, ranging between 52% (ITA) and 100% (FIN) (Fig. 1). Values vary from North to South: in SWE and FIN figures are higher than 90%, in Centre-West European countries (FRA, GER and AUT) figures are higher than 60%, in East European countries (SVK and RUS) figures are higher than 70%, and in South European countries (ITA and ESP) figures are lower than 60%.

The percentage of respondents who have heard about bioeconomy increases from BSc (about 65%) to MSc (76%) and finally PhD (86%).

**Fig. 2:**
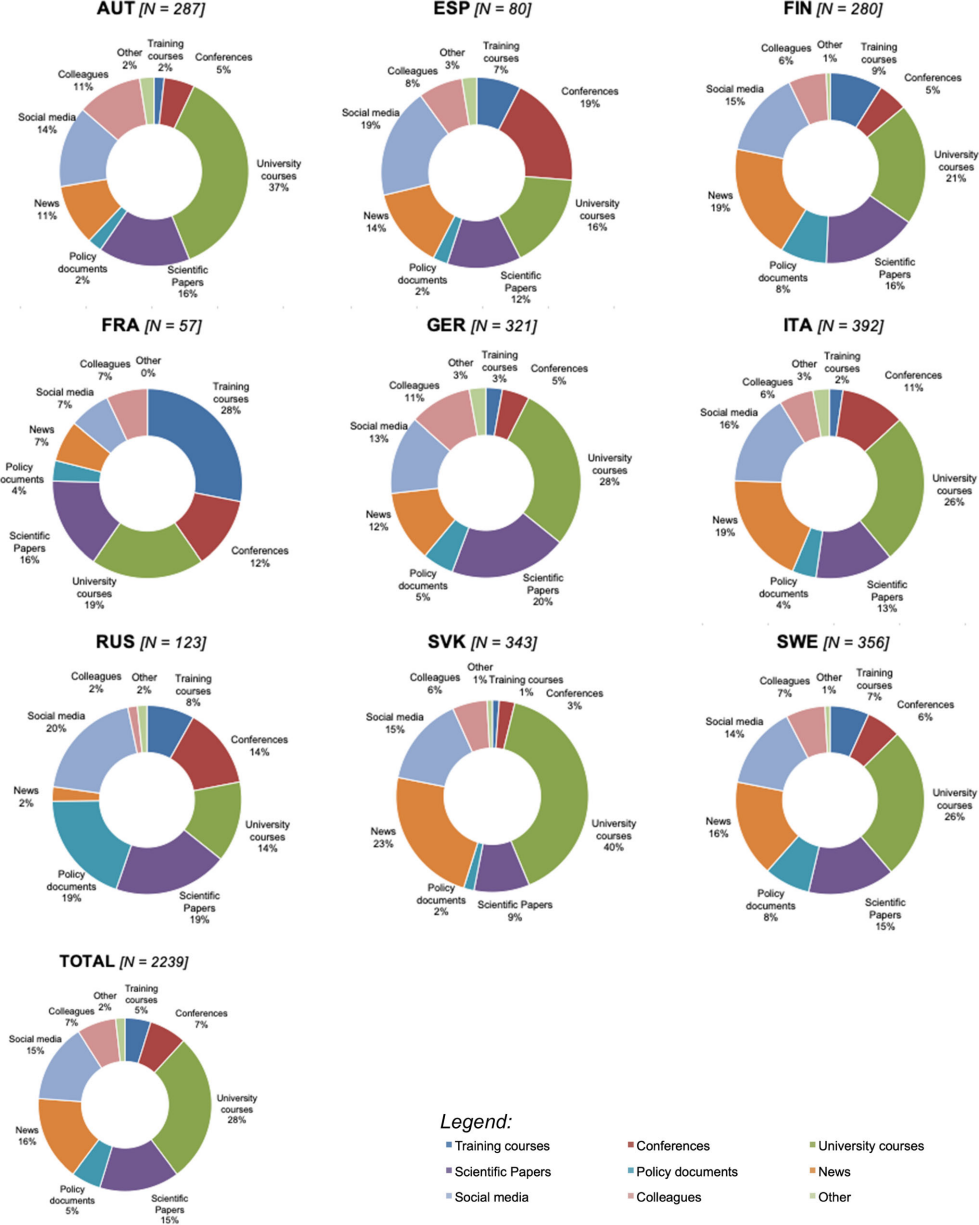
Main information sources on bioeconomy for respondents. *Note* Multiple choices are allowed; therefore, total values do not equal the number of respondents. *AUT* Austria, *ESP* Spain, *FIN* Finland, *FRA* France, *GER* Germany, *ITA* Italy, *RUS* Russian Federation, *SLK*  Slovakia, *SWE* Sweden

The main sources of information about bioeconomy identified by respondents are university courses (28%)—both in total and for six out of nine target countries—followed by news (16%), scientific papers (15%) and social media (15%) and colleagues and conferences (7% each) (Fig. 2).

Among courses that have been reported to address bioeconomy issues, courses in economics are the most cited ones (about 37% of the total courses mentioned), followed by forest management (FM) and silviculture (21%), policy (16%), ecology (14%) and technology (11%).

In order to validate the reply to the question “*have you heard about bioeconomy?*” respondents were asked to show possible awareness of European and national bioeconomy strategies. The “Don’t know” option resulted the most common one (63% and 60% respectively) and although awareness is concentrated (90%) among respondents who have heard already about bioeconomy these remain largely undecided (57% and 47%). Despite some differences among countries, awareness of European/national strategies, as well as the incidence of university courses as a source of information, tend to increase along students’ careers, i.e. from BSc to MSc and finally PhD.

### Cluster analysis

**Fig. 3:**
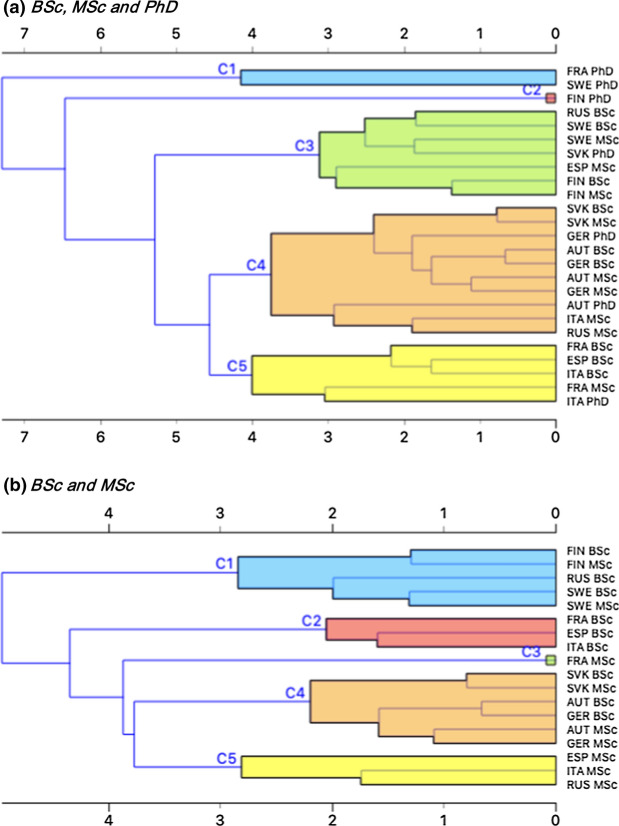
Hierarchical cluster analysis dendrograms showing different clusters identified via 23 variables reported in Table 2 for two different datasets considered: **a** BSc, MSc and PhD and **b** BSc and MSc

Results of the cluster analysis are summarized in Fig. 3 and discussed below.

When the whole dataset is considered (Fig. 3a), four clusters (C1, C3, C4 and C5) and one outlier (C2) are observed. The latter corresponds to FIN PhD that would fuse at much higher distances and do not fit into the analysis. Also C1, including FRA and SWE PhD, is visibly isolated from the rest of the clusters and their merging seems to be quite arbitrary. FRA, FIN and SWE are the countries with the lowest number of PhD respondents and this might explain their isolation. In general terms, the distance between PhD and BSc-MSc is larger (i.e. higher difference) than the distance between BSc and MSc within the same country. The shortest distance (i.e. higher similarity) between BSc and MSc within the same country is observed for SVK, with a convergence inside C4 at a rather low value on the horizontal axis, followed by FIN, AUT, GER and SWE. On the contrary, the gap between ITA, ESP and FRA and RUS BSc and MSc is larger as they converge at higher distances. Convergence is also observed between university programs across countries: this is in particular the case for AUT and GER BSc as well as MSc, forming the bulk of C4, and ESP and ITA BSc (C2). Cross-country convergence is faster for BSC than other groups.

Given the limited number of PhD respondents, their uneven distribution across countries, with a strong concentration in GER and no respondents for ESP and RUS, we decided to focus on BSc and MSc (Fig. 3b). Overall, 4 clusters (C1, C2, C4 and C5) and one outlier (C3, i.e. FRA MSc) have been identified. They are described below, *vis*-*à*-*vis* the different blocks of variables analysed (see Table 2).

#### Bioeconomy within attended university programs

**Fig. 4:**
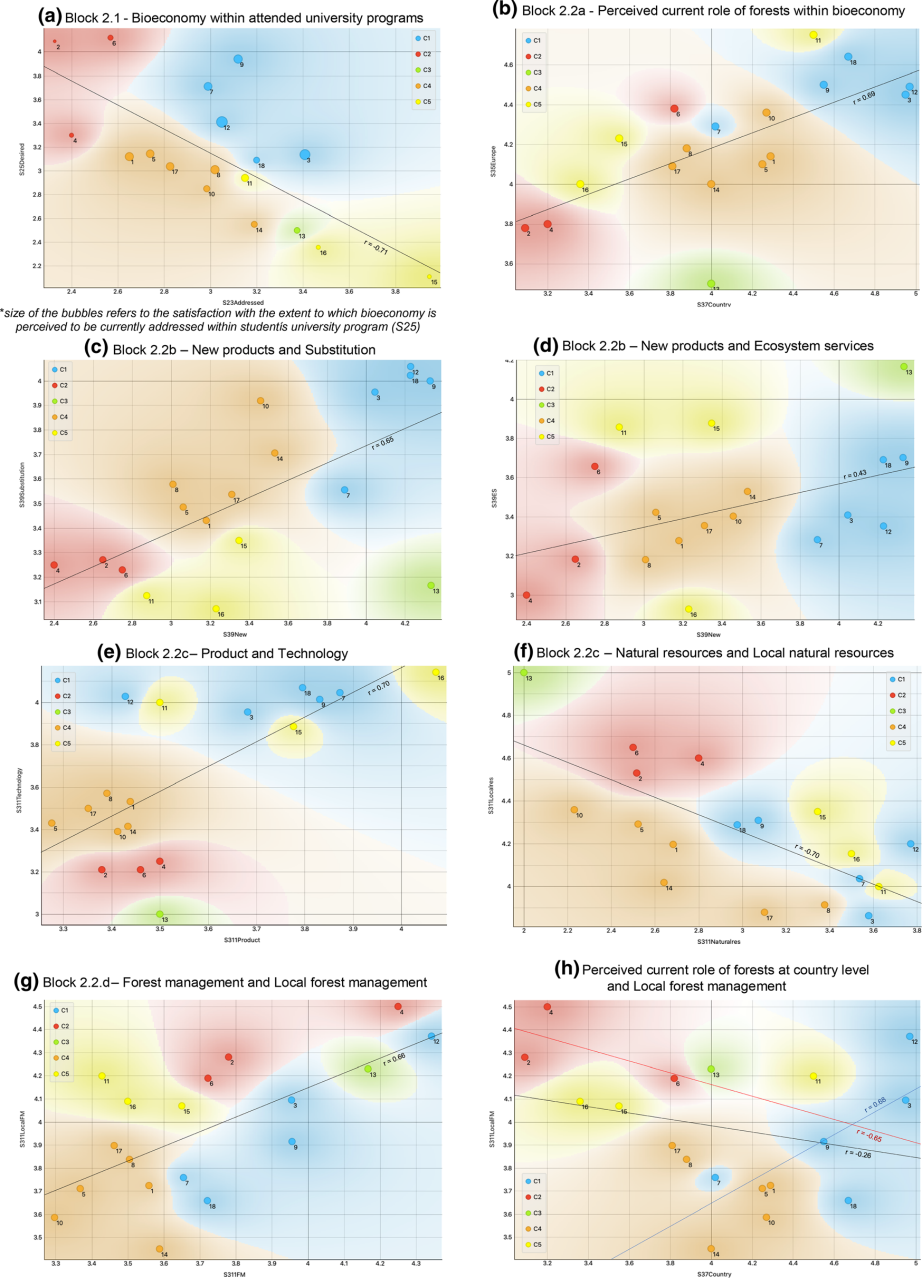
Plot charts of key variables used for the cluster analysis. Note: 1. AUT BSc, 2. ESP BSc, 3. FIN BSc, 4. FRA BSc, 5. GER BSc, 6. ITA BSc, 7. RUS BSc, 8. SVK BSc, 9. SWE BSc, 10. AUT MSc, 11. ESP MSc, 12. FIN MSc, 13. FRA MSc, 14. GER MSc, 15. ITA MSc, 16. RUS MSc, 17. SVK MSc, 18. SWE MSc, 19. AUT PhD, 20. FIN PhD, 21. FRA PhD, 22. GER PhD, 23. ITA PhD, 24. SVK PhD and 25. SWE PhD

*Cluster 1*, C1 (FIN and SWE BSc and MSc, RUS BSc) includes respondents who perceive bioeconomy as moderately addressed within the university programs they attend (Fig. 4a) are little to rather satisfied and ask the bioeconomy is taught from rather more to more. The most satisfied respondents are FIN BSc and MSc.

*Clusters 2* (ESP, FRA and ITA BSc) and *5* (ESP, ITA and RUS MSc) include the least satisfied respondents. *Cluster 2* (C2) associates this with the lowest perceived extent to which bioeconomy is currently addressed and the highest request for more bioeconomy teaching. *Cluster 5* (C5), on the contrary, represents the highest perception of the extent to which bioeconomy is addressed, but also the biggest dissatisfaction of respondents as well as the lowest request for bioeconomy to be taught more.

*Cluster 4* (C4; AUT, GER, SVK BSc and MSc) lays between C1, C2 and C5 and shows intermediate values for all variables: this means that students are little to rather satisfied with the current teaching and that they wish bioeconomy to be a bit more addressed within their programs. C3 (FRA MSc) falls between C4 and C5: despite reporting the second highest value for the perceived extent to which bioeconomy is addressed, it also shows moderate to low levels of satisfaction and students’ requests for more bioeconomy teaching.

BSc tend to concentrate in the upper-left part of Fig. 4a, while MSc tend to concentrate in the lower-right part. As indicated by the larger gap between C1 and C3/C5, if compared to the one within C1 and C4, the within-country BSc-MSc gap is larger for ESP, FRA and ITA than for AUT, FIN, GER, SVK and SWE. The most evident BSc-MSc gap is observed for ITA, while the lowest ones for SVK, FIN, AUT and SWE.

#### The importance of forests within a bioeconomy

C1 includes respondents perceiving the highest values for forests at both country and European level and showing a positive difference (gap) between them (up to +0.5). RUS BSc does not fit very well in C1, as it shows a negative gap. C2 and C5 present the lowest perceived values at country level and the largest negative gaps (− 0.6 to − 0.7) with the importance of forests within a bioeconomy perceived at the European level. ITA BSc (C2) and ESP MSc (C5) present higher values than their cluster members, but still show negative gaps of the same magnitude. C3 and C4 fall between C1, C2 and C5. C3 presents the lowest value for the European level and a positive gap similar to the one observed for FIN. As for C4, AUT and GER BSc show similar values, including the gap (0.2), and AUT and GER MSc have zero gaps (Fig. 4b).

#### The development of a forest-based bioeconomy

With regard *to aspects perceived as developed through FBB nowadays*, C1 shows the highest value for the development of totally new products and technologies as well as the substitution of fossil fuels, followed by improved products and efficiency. C2 presents the lowest values for all variables, in particular with reference to the development of new products and substitution. C5 is similar to C1, but shows higher values for the development of new products (and technologies) and ecosystem services and lower for the fossil fuel substitution. C3 presents values higher than 4 for all variables but substitution, for which it shows values similar to C2 and C5. When compared to C2 and C5, C4 shows higher values for efficiency and substitution, but lower for ecosystem services.

The development of new products and technologies and the substitution of fossil fuels and ecosystem services seem to be the most polarizing issues (Figs. 4c and d). As for substitution, C1 and C4 show medium to high values, standing above all other clusters; C1 and C3 have values higher than 4 for the development of new products (except for RUS BSc) and C3 and C5 have the highest values for ecosystem services, although ITA BSc (C2) and SWE BSc and MSc (C1) show high values too.

With reference to the *perceived FBB development drivers and orientations*, C1 and C5 perceive the highest role for technology as a bioeconomy driver together with the perception that bioeconomy shall be oriented towards products. The remaining clusters show a much lower perceived value for both technology (in particular C2 and C3) and products (Fig. 4e). C2 and C3 highly perceive the FBB to be oriented towards ecosystem services, while all other clusters report lower values. All clusters tend to agree about the use of local resources, as well as the combination of new and traditional knowledge: for the two of them all rates are higher than 4. When considering the use of natural resources regardless of their origin (i.e. no matter if locally or imported), the range of perceptions is much broader: C1, C5 as well as SVK BSc and MSc (C4) are open to the possibility of using non-local resources (moderate agreement). On the contrary, C2, C3 and C4 (excluding SVK) rather disagree with this idea as they seem to be more in favour of local resources (Fig. 4f).

Finally, with reference to the *perception of possible FBB impacts*, (C1), FRA BSc (C2) and MSc (C3) understand the development of a bioeconomy to promote FM both locally and at a broader scale. C2 and C5 are more oriented towards the idea that a FBB will support local FM activities (Fig. 4g). The promotion of local FM via FBB can be linked to the perceived role of national forests within bioeconomy (Fig. 4h). In the case of C1, the development of a FBB might be regarded as an opportunity to further reinforce the local/national forest sector, while in the case of C2, it may provide an opportunity to enhance it. This may also be linked to additional benefits: C2 (as well as C5 and C3) strongly perceive that the development of a FBB will promote employment opportunities.

All clusters tend to agree with the idea that the development of a bioeconomy will favour sustainable forest management (SFM); however, C4 (AUT and GER) shows a fully undecided position.

**Fig. 5:**
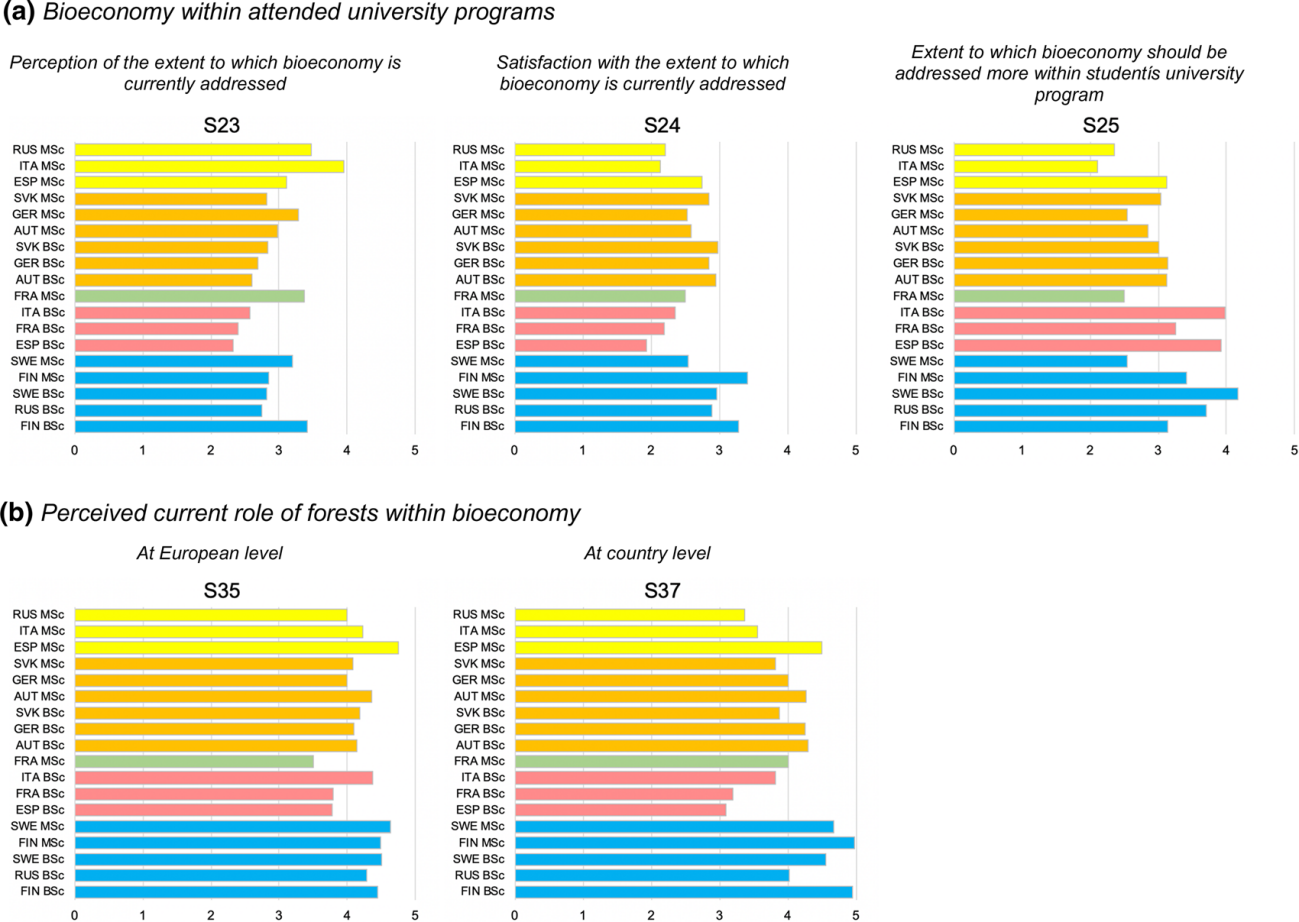
Clustering of perceived values for **a** bioeconomy within attended university programs (variable block 2.1) and **b** perceived current role of forests within bioeconomy (variable block 2.2)

**Fig. 6:**
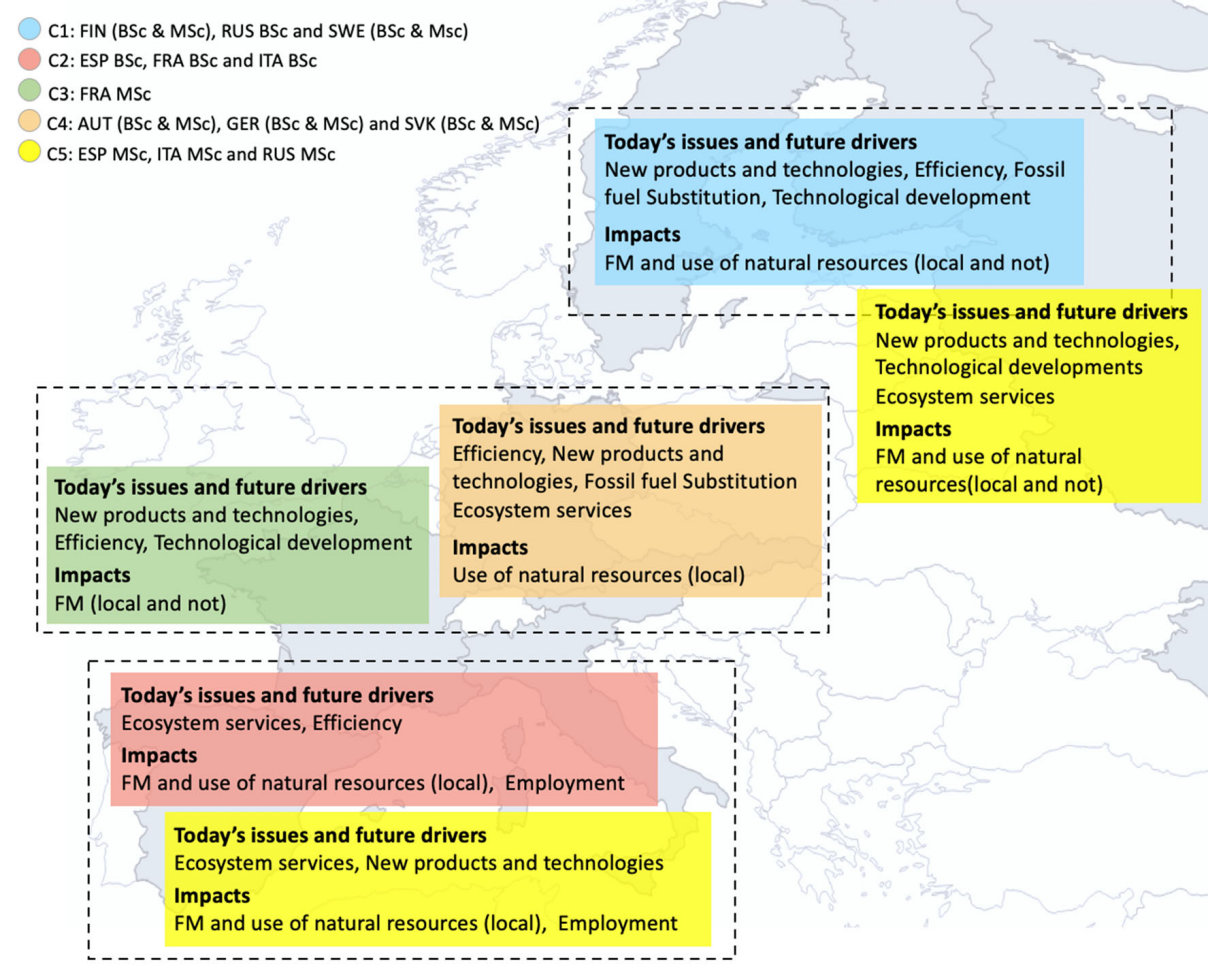
Visual summary and mapping of clustering for perceived FBB today’s issues, future drivers and impacts (variable block 2.3)

The key results of the clustering exercise are reported in Figs. 5 and 6 in the form of a visual summary and mapping of clusters and their main features.

## Discussion

### Bioeconomy: a flowing and waving master narrative

The word “bioeconomy” and related terminology is increasingly mentioned within technical and policy documents and is gaining traction in public and media discourse, as it has been emphatically defined as “*panacea for sustainable competitive growth*” (Philippidis et al. 2016). Not surprisingly, then, about 70% of the respondents reported to have heard about it and this percentage grows up to 100% for forestry students in FIN. Having heard about bioeconomy doesn’t necessarily imply that students completely understand it and are familiar with it. Being familiar includes “*perceiving, interpreting, remembering and responding to stimuli*” (Purdy 1997, p. 8) as necessary steps for processing information and a primary channel of learning processes (Duck and McMahan 2017). Respondents reported university courses to be their main source of information on bioeconomy: this confirms the role of HEI in contributing to a transition towards a bioeconomy as emphasized by several policy documents, including the EC Bioeconomy Strategy (EC 2018). However, since the rise of the importance of a bioeconomy is quite recent, building of an effective education system by HEI for addressing it systematically and critically is still at the early stages. Almost one-third of respondents stated that the bioeconomy concept is a completely new and unknown to them and that they cannot provide a definition. Based on respondents’ feedback, bioeconomy is perceived from different perspectives and with slightly different meanings. Complex terms/concepts, like bioeconomy, tend to be understood only subjectively and are individually interpreted (Golowko et al. 2019). This will likely result in several different developments of the bioeconomy, as confirmed by respondents’ associations focusing on different topics such as technology developments, ecosystem services or substitution. Students’ perceptions confirm findings from previous studies, i.e. the meaning of bioeconomy “*still seems in a flux*” (Pülzl et al. 2014, p. 386) and it can be characterized as a “*master narrative*” (Delvenne and Herndrickx 2013, p. 75; Levidow et al. 2013, p. 95), which is open for very different interpretations and conceptions (Kleinschmit et al. 2014; Pfau et al. 2014; Bugge et al. 2016; D’Amato et al. 2017; Hausknost et al. 2017; Bauer 2018; Vivien et al. 2019).

### More bioeconomy at university: more of what?

Bioeconomy is addressed unevenly across different university programs in Europe and so is its embodying within targeted universities. This reflects on the level of satisfaction with the extent to which bioeconomy is perceived to be addressed: overall about 40% of respondents are not/little satisfied and about 90% of respondents would like to have more bioeconomy taught within their university programs. Clustering allows identifying differences across both countries and university programs. The least satisfied are students from ESP, FRA, ITA and RUS (MSc) while higher satisfaction is reported for all other countries, in particular for FIN. All in all, countries that have been the first (as early as 2011) in Europe to start developing national bioeconomy discourses, such as AUT, GER and FIN, reported bioeconomy to be more addressed and students to be more satisfied.

BSc and MSc reported different perceptions: the former group perceives bioeconomy as being too little addressed and would like to have more bioeconomy-related courses at their home institutions. However, differences regarding perceived satisfaction across BSc and MSc groups are nuanced and country specific.

It is not easy to say how “more bioeconomy within university programs” should be interpreted based only on survey results, because this question was not explicitly asked for. When comparing responses to different questions across the whole questionnaire it can be argued that respondents are asking to learn more about bioeconomy, but also (Section 5 of the questionnaire, not covered here) to have the opportunity to learn about it via a problem-oriented learning that combines basic theoretical concepts acquired during courses with more practical and professional life-oriented approaches.

Few courses dealing with bioeconomy from a social-scientific perspective were identified. This is an important finding, considering that bioeconomy in not purely a techno-scientific or economic concept (Goven and Pavone 2015). In order to understand the complex socio-political phenomena shaping this concept, bioeconomy also has to be taught from a critical, social-scientific perspective. According to Repko et al. (2013) most university programs are I-shaped, i.e. they tend to give students in depth knowledge/expertise within one specific discipline. Bioeconomy is “*multidisciplinary in nature and it pushes us to be interdisciplinary in our approach*” (Geoghegan-Quinn 2010, p. 4). This makes it appropriate for favouring T-shaped profiles for graduates, i.e. profiles combining a deep expertise in a certain field, with integrative abilities allowing to move across different disciplines and link with them (Lask et al. 2018). Bioeconomy demands cross-functional and multidisciplinary knowledge (Hakovirta and Lucia 2019). Considering that forestry is a multidisciplinary domain such a transition should be coherent with its own nature, while at the same time encouraging cooperation with other disciplines and leading towards a better topical integration across BSc and MSc programs. Besides the more “classical” techno-scientific and management-oriented disciplines taught at forestry universities, fundamental knowledge of the industrial value chains, harvesting and supply chains, manufacturing, logistics and trade of bio-based products and bioeconomy-related services is needed (Golembiewski et al. 2015; Hakovirta and Lucia 2019). Lastly, contributions from universities are the cornerstone for the necessary innovation in the bioeconomy (van Lancker et al. 2016): bioeconomy education should foster a culture of innovation.

### The perceived role of forests

Forestry students perceive the forest-based sector to currently contribute to a bioeconomy in Europe and at national level, however differences across countries can be identified. FIN and SWE (C1), AUT and GER (C4) and FRA (MSc) (C3) consider that the contribution to the bioeconomy by forests at the national level is higher than their contribution at the European scale, while for all other countries (and in particular for C1 and C5) the opposite situation is observed. Except for C3, target countries where students declare they are more aware of a bioeconomy, they also perceive bioeconomy is more addressed within their university programs and are more satisfied, are also those where they perceive the forest-based sector to be more important within the national bioeconomy. This reflects the relative importance of the forest sector at national level in terms of its contribution to the gross domestic production (Forest Europe 2015). It is also linked to a prevalently technology-driven and product-oriented view of the FBB, with a primary focus on provisioning services (i.e. biomass production) rather than a FBB interpretation giving emphasis to multiple ecosystem services provided by forests.

The bioeconomy is perceived as an opportunity for the forest sector, rather than a threat. On average respondents agree with the idea that the development of a bioeconomy will favour FM and lead to more SFM. Nevertheless, differences emerge in terms of where this will occur. Respondents from ESP, FRA and ITA expect the bioeconomy will mainly favour FM at the local scale, generating positive impacts also on local communities (e.g. employment opportunities), while others expect the bioeconomy to promote FM in general. AUT and GER (C4) reported some concerns regarding SFM that seem to be consistent with similar considerations related to SFM in a bioeconomy context and existing studies. Stern et al. (2018a) for instance reported respondents from AUT associating bioeconomy with an exploitation of natural resources. The political bioeconomy discourse in the countries that have dedicated bioeconomy strategies is generally dominated by economic goals. In contrast, environmental concerns are only considered to a limited extent (Kleinschmit et al. 2017; Ramicilovic-Suominen and Pülzl 2018). Forest resources are attributed an essential role in the bioeconomy discourse of North European countries (e.g. FIN and SWE) as compared to bioeconomy policies from others (Kleinschmit et al. 2017). These political discourses are well reflected in the student answers from these countries and may become more relevant when considering the need to reconcile bioeconomy policies—looking at forests mainly as biomass sources—and the newly approved EU Biodiversity strategy to 2030—that sets targets for more nature conservation—within the framework of the EU Green Deal.

### Routes to bioeconomy development

By comparing survey results and clustering to existing literature, different FBB development routes can be identified. Although many nuanced situations can be observed, both across and within countries, a continuum of different visions and transition paths (TP) appears (Table 3). Bioeconomy perception by forestry students within C1 (in particular FIN and SWE) partly overlaps with the “Life science vision” described in Levidow et al. (2013) and Vivien et al. (2019) and falls between the “sustainable capital” (Birch et al. 2010) and “planned transition” visions identified by Hausknost et al. (2018) and between the “bio-technology” and the “bio-resource” visions reported in Bugge et al. (2016). It can be described as a technology-led shift to bio-innovations, following a linear innovation approach (Rametsteiner and Weiss 2006; Hansen et al. 2011), where the ecologically sustainable use of resources is achieved via advanced (bio)technologies applied at (large scale) industrial level (Hausknost et al., 2018). Such a perspective seems quite in line with the dominant paradigm according to which forest bioeconomy and its innovations in Europe are mainly technologically oriented (Lovrić et al. 2018) and industry dominated (Schmid et al. 2012). The key-driving force at play is decarbonisation with economic growth and competitiveness among the main aims. The prevalent TPs towards a bioeconomy are those of substitution and productivity increase, followed by efficiency increase (Dietz et al. 2018). This position reflects ideas behind the corresponding bioeconomy national strategies and their development approaches (Staffas et al. 2013; Dubois and Gomez San Juan 2016; Pülzl et al. 2017; Hausknost et al. 2018). As regards C4 (in particular AUT and GER), although bio-based production and technology remain paramount, the focus is on efficiency that, within a FBB perspective, is translated into sustainable intensification (Godfray et al. 2010), cascading approach (Keegan et al. 2013), wood-waste reduction and circularity. Efficiency and productivity increase are therefore the main TP, while a shift in visions towards in-between bio-resource/bio-technology (Bugge et al. 2016), sustainable capital/eco-growth (Hausknost et al., 2018) and type II and III visions (i.e. science-based and biomass-based economy) (Vivien et al. 2019) positions is observed among the respondents’ answers to the survey. Research and development, knowledge, and technology are relevant factors at play, and linear innovation remains the main paradigm, however the focus is not just on bio-based products and some attention is paid to a broader range of forest ecosystem services.

**Table 3:** FBB development routes based on student’s perceptions vis-à-vis existing literature

	Northern Europe	Central Europe	Southern Europe
Countries	FIN and SWE (RUS)	AUT and GER (SVK, FRA MSc)	ITA and ESP (FRA BSc)
Clusters	C1	C4 (C3)	C2 (C5)
Today’s issues and future drivers	New products Efficiency Fossil fuel Substitution Technology	Efficiency New products Fossil fuel Substitution Technology Ecosystem services	Ecosystem services New products
Impacts (see Figs. 5 and 6)	FM and use of natural resources (local and not)	Use of natural resources (local)	FM and use of natural resources (local) Employment
Bioeconomy visions a. Bugge et al. (2016) b. Hausknoft et al. (2018) c. Vivien et al. (2019)	a. Bio-technology/Bio-resource b. Sustainable Capital/Planned Transition c. Life science, Type II and III	a. Bio-resource/Bio-technology b. Sustainable Capital/Eco-Growth c. Type II and III	a. Bio-resource/Bio-Ecology b. Eco-Growth/Eco-Retreat c. Type I and III
Transition paths (TP), decreasing importance/prevalence (Dietz et al. 2018)	TP1-Substitution TP2-Productivity increase TP3-Efficiency increase TP4-Value creation	TP3-Efficiency increase TP2-Productivity increase TP1-Substitution TP4-Value creation	TP4-Value creation TP3-Efficiency increase TP1-Substitution TP2-Productivity increase
Innovation process Rametsteiner and Weiss (2006) Hansen et al. (2011) Secco et al. (2018)	Mainly linear	Mainly linear with some interactive components	Mainly interactive with some linear components
Perceived current importance of national forests within transition to a FBB (see Fig. 4)	Very high	High	Medium/Low

*Variable values have been obtained from survey data by averaging values per country and attended program (i.e. BSC, MSc and PhD). See additional material available in S1 for more details and referenced questions

C2 and C5 (in particular ITA and ESP, and, to a lower extent, FRA) tend to emphasize bioeconomy components associated to ecosystem services rather than being just bio-product and technology focused. This perception has some affinity with the bio-ecology vision offered by Bugge et al. (2016), the eco-retreat vision by Hausknost et al. (2018) and type I vision (i.e. ecological economy) by Vivien et al. (2019). It suggests moving away from forests being mainly biomass sources to recognizing and mobilizing the “*entire spectrum of ecosystem services that Europe’s forests can provide for the benefit of Europe’s societies*” (Winkel 2020, p. 153). This is connected to the promotion of local FM and rural development opportunities driven by a diversification into higher value-added products and services with territorial identity (Levidow et al. 2013). Value creation is the main TP, followed by an efficiency increase. While this perspective does not imply that bio-products and technology are totally neglected, it suggests moving away from a purely linear technological innovation into a more complex, non-linear innovation process that involves several interactions among different sectors and actors (Rametsteiner and Weiss 2006; Secco et al. 2018).

## Conclusions

By combining data collection via an explorative survey, descriptive statistics, cluster analysis and discussion *vis*-*à*-*vis* existing literature, this paper presented key findings of a study investigating bioeconomy perceptions by forestry students across Europe. These new insights helped us gain a better understanding of forestry students’ perceptions of bioeconomy which may ultimately aid the future development of HEI programs in forestry and serve as important reference for evidence-based educational policies in the future.

The research identified two main perception axes: a major geographical (South–North) and a minor student career (BSc–MSc) axis along which gradients of many surveyed perceptions can be located.

The student career axis seems to be particularly relevant with reference to whether respondents have heard about bioeconomy and perceptions regarding how bioeconomy is currently addressed within university programs. The small sample size of PhD students and the fact that they are mainly concentrated in one country (GER) do not allow drawing general conclusion for this category.

Along the geographical axis, different perceptions are detected with reference to the importance of the forestry sector. The findings show that there are different visions, understandings as well as degrees of maturity and development of the concept as perceived by forestry students and this implies also different development routes towards the transition to a bioeconomy in European countries. The complexity of such a concept, as well as the debates and controversies in scientific discourses about it imply that universities must reflect their position within the societal transformation process, taking into account both the specificities of the national/local environmental and socio-economic contexts and global challenges as well as trends. This seems to suggest a combination of measures that call for integrated viewpoints especially across life science universities, but also outside as bioeconomy strategies are so-called integrated strategies that are important for everyone. However, integration does not stand in for harmonization, but rather for a cooperative approach. This should start by *engaging and creating awareness*: since only one-fourth of respondents are aware of EU/national bioeconomy strategies, this awareness and knowledge gap should be filled. Measures could start providing basic information and a general framework, directions and aims for a bioeconomy development. Questions arise regarding not only the capacity to reach out to future stakeholders, during the development of policy strategies, but also after their publication. Failing to effectively engage future stakeholders at this stage and in communicating and creating awareness might nourish conflicts for the future development of a bioeconomy. Integration should also be sought within and across HEI, as well as between HEI and the “outside world”. *Integration within HEI* should foster interdisciplinarity within programs, bridging BSc and MSc curricula where large gaps have been identified with regard to bioeconomy teaching (e.g. ITA, FRA and ESP) and filling the perceived gap in policy courses dealing with bioeconomy. Students could be given a broader interdisciplinary perspective of the bioeconomy concept first and then be allowed to choose on which aspects to focus on.

Integration across HEI may help coordinating how bioeconomy is addressed in different countries and favour the exchange of different perspectives and approaches to this concept. This could be done both within the same cluster, i.e. involving HEI that supposedly share similar visions and approaches, to help cooperation and advance research and knowledge, and across different clusters, to support spreading of different views and bioeconomy development paths. Recently created programs and university consortia focused on bioeconomy, as well as more traditional student exchange programs might serve this purpose and could be integrated with other initiatives spanning from education (including online and blended courses on specific bioeconomy-related topics) to cross-boundary research projects (Lovrić et al. 2020).

Finally, integration of HEI and the “world outside” would require reinforcing the role of the “third mission” in universities, i.e. the generation and transfer of knowledge outside the academia by strengthening dialogue and interaction among university and non-university actors. This could help actively engaging future bioeconomy stakeholders within the debate and challenge them with real-world problems while at the same time integrating HEI programs.

How to practically develop the above-reported integration options and the extent to which they can contribute improving HEI programs addressing bioeconomy, however, needs to be further explored.

Despite our efforts to cover perceptions from different European regions, this study is far from exhaustive. The uneven country samples, and the selection of (mainly) one university within each country represent two limiting research aspects that might be integrated in future research activities, although we are aware that enlarging the study scope would be resource intensive. Possible additional research might include running similar surveys within students attending different programs (e.g. in agriculture, economics, energy engineering, materials engineering etc.) at targeted universities/countries, to compare views and perceptions form different domains. The questionnaire might be revised and shortened, thus reducing fatiguing effects reported by some of the respondents.

This study has shed light on how European forestry students perceive the bioeconomy and their expectations from its development. It has shown that perceptions vary across European regions and that much work remains to be done in terms of synchronizing educational efforts and for adapting the curricula for the growing demand for cross-functional and interdisciplinary bioeconomy education.

## Electronic supplementary material

Below is the link to the electronic supplementary material.

Supplementary material 1 (PDF 1351 kb)
