# Supplementary material for: Bioeconomy perception by future stakeholders: Hearing from European forestry students
Source: Ambio. 2020 Oct 13;49(12):1925–42. doi: 10.1007/s13280-020-01376-y (PMC7568748; doi:10.1007/s13280-020-01376-y)
Supplement: Supplementary file 1 — Supplementary material 1 (PDF 1351 kb) [file 13280_2020_1376_MOESM1_ESM.pdf]

Ambio

Electronic Supplementary Material

This supplementary material has not been peer reviewed

Title: **Bioeconomy perception by future stakeholders: Hearing from European forestry students**

Mauro Masiero, Laura Secco, Davide Pettenella, Riccardo Da Re, Hanna Bernö, Ariane Carreira, Alexander Dobrovolsky, Blanka Giertliová, Alexandru Giurca, Sara Holmgren, Cecilia Mark-Herbert, Lenka Navrátilová, Helga Pülzl, Lea Ranacher, Alessandra Salvalaggio, Arnaud Sergent, Juuso Sopanen, Cristoph Stelzer, Theresa Stetter, Lauri Valsta, Jozef Výbošťok, Ida Wallin

Appendix S1. Questionnaire used for the survey on bioeconomy perception by future stakeholders

Your opinion about the forest-bioeconomy matters

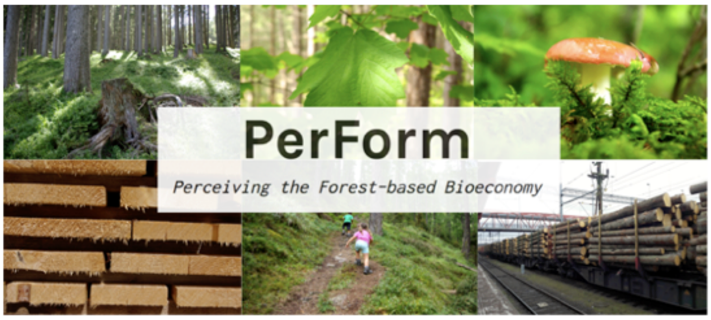

Welcome to our survey on forest-based bioeconomy perceptions.

As a forestry student, you are a key-future stakeholder for the forest-based bioeconomy. This is why your opinion is so important. We are interested in knowing more about how you perceive bioeconomy today and in a future perspective.

All forestry students –including Bachelor, Master and Doctorate ones– from Italy, Germany, France, Austria, Finland, Sweden, Slovakia and Russia can participate in this survey.

This survey consists of an online questionnaire structured into 6 sections –focusing on your knowledge and perception of some key-topics, to perspectives in terms of future job opportunities– and will take about 20 minutes of your time. Your responses will be later analysed and compared with those of your colleagues from across Europe.

Want to know more about the project?

PerForm is a European cooperation network supported by the European Forest Institute (EFI). We are an international team of social scientists from eight top-ranked European research institutions, investigating societal perceptions of the forest-based bioeconomy.

PerForm aims to better understand regional disparities of national bioeconomy policies and explore the diversity of perceptions and acceptance of a forest-based bioeconomy by different forest stakeholders across Europe and Russia. For more information: https://perform-bioeconomy.info

PerForm partners:

External cooperation institution:

Privacy notice

Data collected through this survey will be treated confidentially and anonymously for the purposes of PerForm project research, in compliance with the General Data Protection Regulation (GDPR), Regulation (EU) 2016/679.

By filling the questionnaire, you give PerForm network staff the permission to process data you provide for the purposes of PerForm project research.

S0 - Select your language

Please select your language from the menu or select additional languages through links given below:

S1 – How familiar are you with bioeconomy?

S11 - Have you ever heard about bioeconomy or bio-based economy?
[*Yes; No]*

S12 - If yes, where?

- Training courses
- Conferences
- University courses
- Scientific Papers
- Policy documents
- News
- Social media
- Colleagues
- Other:

S13 - How would you define bioeconomy, according to your personal understanding?

[*Max 50 words*]

S14 - To your best knowledge, does the European Union have a specific strategy for bioeconomy?
[*Yes; No; I do not know]*

S15 - To your best knowledge, are you aware of a national or an industry specific strategy for bioeconomy in your home country?
[*Yes; No; I do not know*]

S2 - Bioeconomy at university

The European Commission defines bioeconomy as follows:

"*Bioeconomy encompasses the production of renewable biological resources and the conversion of these resources and waste streams into value added products, such as food, feed, bio-based products and bioenergy. Its sectors and industries have strong innovation potential due to their use of a wide range of sciences, enabling and industrial technologies, along with local and tacit knowledge*".

S21 - Within your university course, bioeconomy is:

- Presented as an epistemological starting point for the entire program being a back bone in the program itself
- Addressed in one or more dedicated modules
- Addressed in one or more dedicated parallel initiatives (e.g. workshops, seminars, conferences…)
- Mentioned in one or more non-dedicated modules/teachings
- Not addressed or mentioned in any modules/teaching
- I do not know

S22 - In your current program, what course has offered beneficial learning conditions to promote the understandings of forest-based bioeconomy? Please enter the course name

S23 - Overall to what extent do you think bioeconomy is addressed within your university curriculum? (note: please consider all credited courses you have been able to take in your program)
[*1= not at all, 2= seldom, 3= sometimes, 4= often, 5= very often*]

S24 - How much are you satisfied with the extent to which bioeconomy is currently addressed within your university course?
[*1= not satisfied, 2= little satisfied, 3= rather satisfied, 4= satisfied, 5=very satisfied*]

S25 - Do you think it is necessary to address bioeconomy more in your University's curricula?
[*1= no, 2= yes, a little bit, 3= yes, rather more, 4= yes, more, 5= yes, very much more*]

S26 - Are you currently developing/planning to develop a thesis dealing with bioeconomy issues?
[*Yes; No*]

S27 - If yes, please enter the (expected) title and/or the main topics

S3 - Activities, issues, sectors and actors associated to bioeconomy

S31 - In your opinion, to what extent do these sectors contribute to bioeconomy in **Europe**?
[*1= not at all, 2= seldom, 3= sometimes, 4= often, 5= very often*]

- Agriculture
- Bioenergy and biofuels
- Building and constructions
- Chemistry
- Education
- Feed
- Fishery and aquaculture
- Food and beverages
- Forestry
- Livestock
- Pharmaceutical
- Pulp and paper
- Textile
- Tourism and recreation

S32 - Do you think there is any other sector that should be included within the above-reported list? Please enter those sector(s)

S33 - In your opinion, to what extent do these sectors contribute to bioeconomy in the **country where your academic program is offered**?
[*1= not at all, 2= seldom, 3= sometimes, 4= often, 5= very often*]

- Agriculture
- Bioenergy and biofuels
- Building and constructions
- Chemistry
- Education
- Feed
- Fishery and aquaculture
- Food and beverages
- Forestry
- Livestock
- Pharmaceutical
- Pulp and paper
- Textile
- Tourism and recreation

S34 - Do you think there is any other sector that should be included within the above-reported list? Please enter those sector(s)

S35 - In your opinion, how important is the **current** role of forests within bioeconomy in **Europe**?
[*1= not important, 2= rather not important, 3= undecided, 4= rather important, 5= important*]

S36 - Please motivate your choice by reporting the main reasons/arguments for attributing such a role

S37 - In your opinion, how important is the **current** role of forests within bioeconomy in the **country where your academic program is offered**? [*]
[*1= not important, 2= rather not important, 3= undecided, 4= rather important, 5= important*]

S38 - Please motivate your choice by reporting the main reasons/arguments for attributing such a role

S39 - According to various studies, bioeconomy could contribute developing forestry. In your opinion, to what extent are the following aspects/issues developed through bioeconomy nowadays?

[*1= not at all, 2= seldom, 3= sometimes, 4= often, 5= very often*]

- Totally new products and technologies (e.g. biochemicals, nanocellulose…)
- Improvement of existing products (e.g. engineered wood products for the building sector)
- Efficient use of forest-based products
- New uses for existing products (e.g. cellulose/cork used in the building sector)
- Substitution of fossil fuels with forest biomass for energy purposes
- Valuing of multiple services/products offered by forests (e.g. ecosystem services)

S310 - Is there any other relevant aspect/issue you would like to include in addition to those listed within S39 above? Please indicate aspect(s)/issue(s)

S311 - As part of the natural capital, forests are largely expected to play a central in bioeconomy development. This is why we can specifically speak about forest-based bioeconomy, i.e. that specific branch of bioeconomy that relies on forest resources.

To what extent do you agree/disagree with the following statements?
The development of a forest-based bioeconomy:
[*1= disagree, 2= rather disagree, 3= undecided, 4= rather agree, 5= agree*]

- Shall be driven by technological developments
- Shall be oriented to products
- Shall be oriented to services (e.g. ecosystem services)
- Shall be based on local resources
- Shall be based on nature resources, no matter if they are local or imported
- Shall try to combine new and traditional knowledge
- Will promote employment opportunities
- Will favour sustainable forest management
- Will promote forest management at local scale
- Will promote forest management, no matter at which scale
- Will lead to increased deforestation/forest degradation
- Will increase people's awareness of environmental and forestry issues

S312 - In your opinion, which are the most relevant key-words for bioeconomy? [*] Please select the top **3 key-words** for bioeconomy within the list given-below.
(at most 3 answers shall be selected)

- Biorefineries
- Decarbonisation
- Ecosystem services
- Efficiency
- Energy
- Industry
- Innovation
- Products
- Rural development
- Social capital
- Technology
- Other:

S313 - Whom do you expect to **benefit/gain** from the development of a forest-based bioeconomy?
[*1= no benefit/gain at all, 2= little benefit/gain, 3= moderate benefit/gain 4= high gain/benefit, 5 = very high benefit/gain*]

- Small private forest owners
- Big private forest owners
- Public forest owners
- Forest enterprises
- Pulp and paper mills
- Sawmills
- Wood industries
- Other industries using forest products as inputs (e.g. energy, textile…)
- Forest product traders
- Consultants
- Financial actors (e.g. investment funds)
- Society at large

S314 - Whom do you expect to **loose/suffer** from the development of a forest-based bioeconomy?
[*1= no loss at all, 2= some loss, 3= moderate loss, 4= high loss = very high loss*]

- Small private forest owners
- Big private forest owners
- Public forest owners
- Forest enterprises
- Pulp and paper mills
- Sawmills
- Wood industries
- Other industries using forest products as inputs (e.g. energy, textile…)
- Forest product traders
- Consultants
- Financial actors (e.g. investment funds)
- Society at large

S4 - Problems and opportunities

S41 - In your opinion, what are the main drivers associated with a transition to a forest-based bioeconomy? Please select the **top 3 drivers** for bioeconomy within the list given-below.

Please select 3 answers
- Climate change
- Substituting non-renewable (e.g. fossil-fuel based) products/materials
- Need for sustainability
- Population increase
- Economic development
- Natural resource depletion
- Need to find new markets and products
- Regulations: national/international policy
- Technology developments
- Environmental degradation
- Generational increase in awareness of sustainable development
- Social medias' role in shaping norms
- Sustainable development standards
- Other (Please enter):

S42 - In your opinion, what are the main problems/barriers associated with a transition to a forest-based bioeconomy? Please select the **top 3 problems/barriers** for bioeconomy within the list given-below.

Please select 3 answers
- Societal disconnection from nature – urbanisation
- Bureaucracy (turning forest utilisation complex and difficult)
- Unclear regulations or policy guidance
- Costs
- Normative resistance
- Competing economic interests and sectors
- Forests as limited resources
- Other:

S43 - In your opinion, what are the main opportunities associated with a transition to a forest-based bioeconomy? Please select the **top 3 opportunities** for bioeconomy within the list given-below.

Please select 3 answers
- To increase society's awareness on environmental issues
- To promote/value the forest sector
- To support an efficient use of forest resources
- To make the forest sector more accepted by the public opinion
- To show the forest sector as part of a greener future
- To create more job opportunities

S5 - Bioeconomy: looking forward

S51 - What kind of job would you like to have once you are finished with your university course? Please enter a job description

S52 - Do you think the development of a forest-based bioeconomy can help you finding such a job?
Please choose one of the following answers

- Yes
- No
- I do not know

S53 - Do you think the development of a forest-based bioeconomy can help creating more job opportunities in general?
Please choose one of the following answers

- Yes
- No
- I do not know

S54 - In your opinion, what are the priorities that should be defined/given to support employment opportunities within the development of a forest-based bioeconomy? Please report the three top-priorities

- Priority 1
- Priority 2
- Priority 3

S6 - General info on respondent

S61 - Age
Only numbers may be entered in this field.

S62 – Gender
- Men
- Female
- No Answer

S63 - Nationality

S64 - Currently enrolled in the following university program (please choose one option)
Choose one of the following answers

- BSc
- MSc
- PhD
- Other (please enter):

S65 - Semester of attendance
Please choose one of the following answers

- 1st semester
- 2nd semester
- 3rd semester
- 4th semester
- 5th semester
- 6th semester
- Other (please enter):

S66 - Institution. Please enter your hosting institution name: University and School/Department/Faculty.

S67 - Are you an Erasmus student or are you taking part in another exchange program (Joint Study, CEEPUS, etc.)?
Please choose one of the following answers

- Yes, Erasmus
- Yes, Erasmus Mundus

- No
- Other:

End of questionnaire

Thanks for participating to the survey: by filling-in the questionnaire and sharing your views you help us gaining a better understanding of future stakeholders' perception and knowledge on forest-based bioeconomy in the EU.

If you want to know more about the survey follow-ups, check for PerForm project updates and look for current and future research opportunities, follow us on https://perform-bioeconomy.info

Stay tuned!

PerForm team

Appendix S2. Number of respondents per target-country and university/institution

Country	University/Institution	N. of Respondents	% on Total
Austria (AUT)	University of Natural Resources and Life Sciences (BOKU)	***216***	***15.8%***
Finland (FIN)	University of Eastern Finland	7	0.5%
	University of Helsinki	54	3.9%
	SUB-TOTAL	***61***	***4.5%***
France (FRA)	AgroParisTech	3	0.2%
	National School of Technologies and Wood Industries	9	0.7%
	University of Tours	3	0.2%
	University of Bordeaux	5	0.4%
	N/A*	1	0.1%
	SUB-TOTAL	***21***	***1.5%***
Germany (GER)	Eberswalde University for Sustainable Development	2	0.1%
	University of Freiburg	213	15.6%
	University of Göttingen	8	0.6%
	University of Hamburg	1	0.1%
	University of Applied Forest Sciences Rottenburg	14	1.0%
	SUB-TOTAL	***237***	***17.3%***
Italy (ITA)	Polytechnic University of Marche	2	0.1%
	University of Bari	1	0.1%
	University of Bologna	2	0.1%
	University of Bolzano	8	0.6%
	University of Firenze	12	0.9%
	University of Molise	3	0.2%
	University of Napoli (Federico II)	4	0.3%
	University of Torino	6	0.4%
	University of Padova	284	20.8%
	University of Viterbo	2	0.1%
	N/A*	4	0.3%
	SUB-TOTAL	***329***	***24.0%***
Russian Federation (RUS)	Primorsky State Agricultural Academy	*6*	0.4%
	St. Petersburg State Forest Technical University	*58*	4.2%
	N/A	*17*	1.2%
	SUB-TOTAL	***81***	***5.9%***
Slovakia (SVK)	Technical University in Zvolen	**225**	***16.4%***
Spain (ESP)	University of Lleida	64	4.7%
	University of Valladolid	4	0.3%
	SUB-TOTAL	***68***	***5.0%***
Sweden (SWE)	Linnaeus University	7	0.5%
	Swedish Agriculture University (SLU)	118	8.5%
	N/A	5	0.4%
	SUB-TOTAL	***130***	***9.5%***
**Total**		***1 368***	***100.0%***

AUT = Austria; ESP = Spain; FIN = Finland; FRA = France; GER = Germany; ITA = Italy; RUS = Russian Federation; SVK = Slovakia; SWE = Sweden.

*N/A = not reported
